# Morphology-matching-based R-wave detection for noise-robust ECG gating

**DOI:** 10.1186/1532-429X-18-S1-P21

**Published:** 2016-01-27

**Authors:** Takami Yoshida, Taketo Kawakami, Sojuro Kato, Hidenori Takeshima, Makoto Hirohata, Shigehide Kuhara

**Affiliations:** 1 Corporate Research & Development Center, Toshiba Corporation, Kawasaki, Japan; 2 MRI Systems Division, Toshiba Medical Systems Corporation, Otawara, Japan; 3 Center for Medical Research and Development, Toshiba Medical Systems Corporation, Otawara, Japan

## Background

Accurate ECG R-wave detection is crucial for cardiac gating in MRI. However, in high-field MRI systems, it is hard to detect R-waves in ECG signals accurately, because the amplitude of the ECG signal may be smaller than that of the noise induced by the MRI system. To overcome this issue, existing studies have focused on (a) acquiring additional ECG signals or on (b) improving the R-wave detector to be robust against noise. In the first approach, ECG gating with a 12-lead ECG has been reported to have high accuracy [1]. However, due to ECG monitor limitations, this study utilizes a common dual-lead ECG. We propose a new morphology-matching-based R-wave detector for noise-robust ECG gating. The morphology is analyzed in filtered ECG signals, and the R-wave is detected by matching the input ECG signals to R-wave templates. The templates are updated when the MRI system is not scanning, and they contribute to robustness against patient variation and noise [1].

## Methods

**Figure 1:**
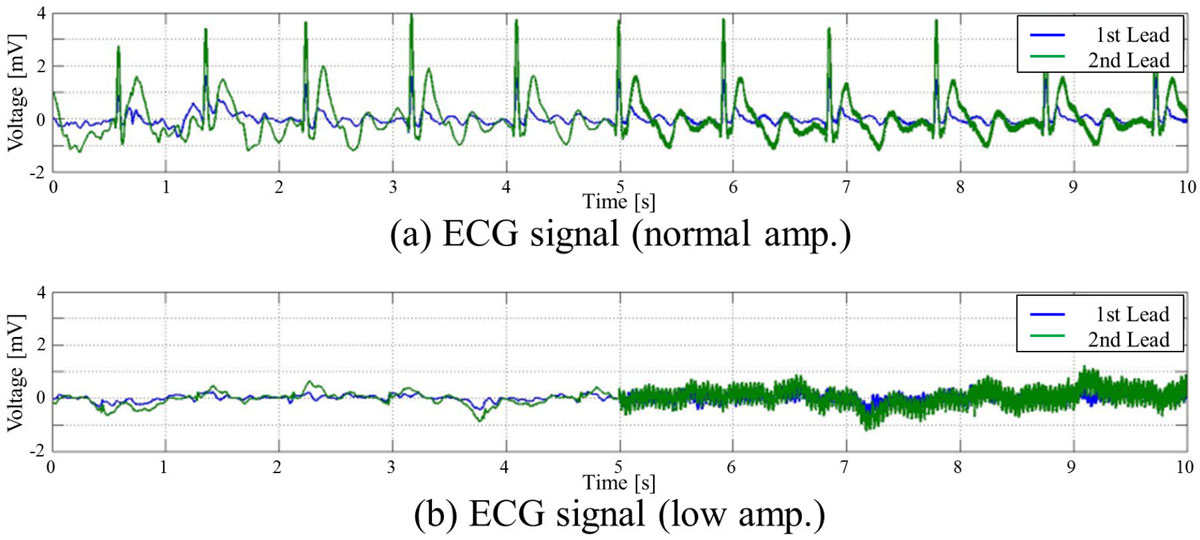
**Examples of the recorded ECG signals**.

Dual lead ECG signals were recorded using a 3-T MRI system (Vantage Titan 3T, Toshiba Medical Systems Corporation) and an attached ECG monitor. We scanned a total of 27 volunteers, including 5 volunteers with low-amplitude ECG waveforms (R-waves are less than 1[mV]). Fig. 1 shows examples of the recorded ECG signals. Each volunteer was scanned using 9 cardiac MRI sequences (Locator, Shimming, Map, axial multislice SSFP, Cine, T2WI, T1WI, perfusion, and LGE). The lengths of the ECG records of the volunteers ranged from 850 s to 1960 s. The proposed R-wave detector processes ECG records using 4 modules: a filtering module, a first stage detection module, a template update module, and a second stage detection module. The filtering module accentuates the R-wave-related frequency components. The first stage detection module detects the peaks from the filtered ECG signals and sends the detected peaks to the template update module. The template update module updates the R-wave templates when the MRI system is not scanning. The second stage detection module then detects the R-waves using the adaptively updated templates.

## Results

**Figure 2:**
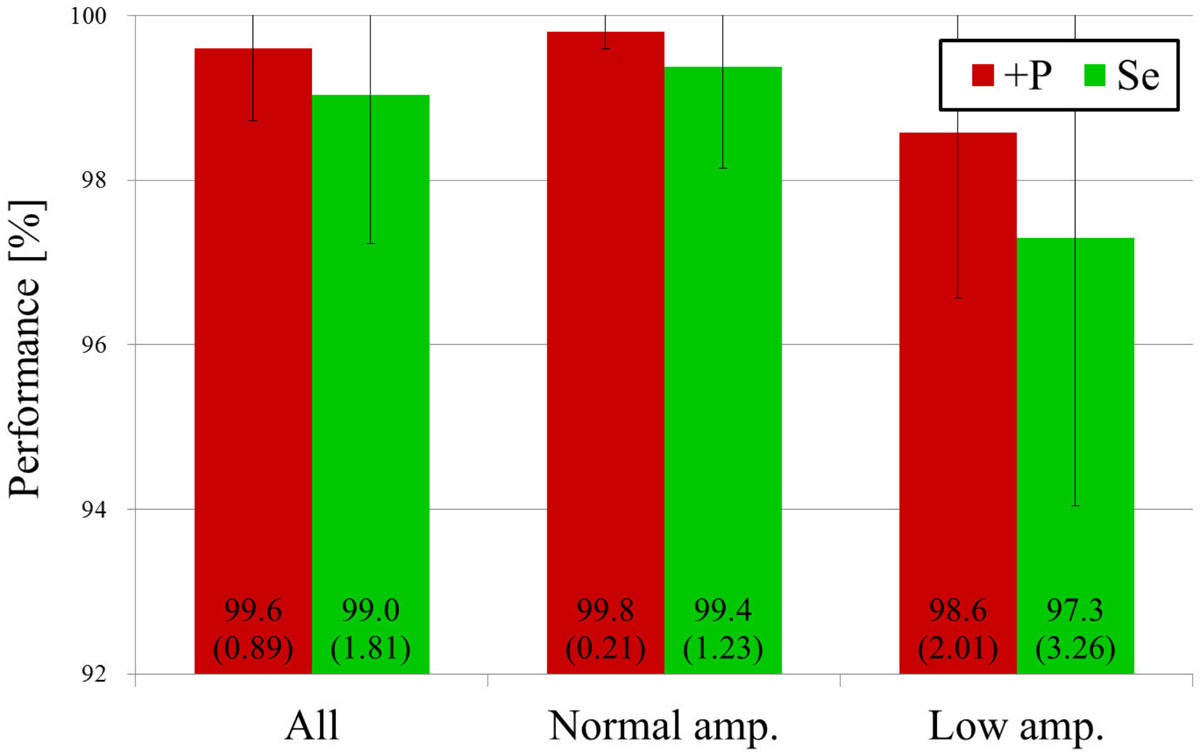
**ECG gating performance (averages and standard deviations) for the proposed technique**.

The proposed R-wave detector was evaluated using the records of above-mentioned 27 volunteers. Fig. 2 shows the positive predictive value (+P) and sensitivity (Se) for the proposed detector. As shown in Fig. 2, +P is higher than Se because the detection of an R-wave overlapped by noise is difficult. To improve Se further, noise suppression technique is promising.

## Conclusions

The proposed method showed that R-wave detection using dual lead ECG can achieve +P of 99.6% and Se of 99.0%. This method is expected to be clinically useful in cardiac MRI examinations, especially in MRI systems with field strengths of 3 T or more.
